# Assessment of Public Knowledge and Awareness on First-Aid Management of Epistaxis in Qatif Region, Saudi Arabia

**DOI:** 10.7759/cureus.78323

**Published:** 2025-02-01

**Authors:** Husam Amoudi, Mazen S AlAithan, Ahmed H AlTheeb, Hamzah Alhajuj, Mohammed M Aljarameez, Ali K Abuhasan, Ali A Ahbail

**Affiliations:** 1 Department of Otolaryngology - Head and Neck Surgery, University of Jeddah, Jeddah, SAU; 2 Department of Otolaryngology, Dammam Medical Complex, Dammam, SAU; 3 College of Medicine and Surgery, University of Jeddah, Jeddah, SAU

**Keywords:** awareness, cross-sectional studies, epistaxis, nosebleed, saudi arabia

## Abstract

Background

Epistaxis, or nosebleeds, is a common condition that can range from minor to severe, with the potential for life-threatening complications. In Saudi Arabia, particularly in Al-Qatif, limited research has been conducted on public knowledge and practices regarding first aid for epistaxis. Despite the condition's frequency, misconceptions and inadequate management techniques persist. This study aims to assess the awareness and attitudes of the general population in Al-Qatif towards first aid for epistaxis.

Methods

This cross-sectional study was conducted in Al-Qatif, Saudi Arabia, from November to December 2024. A self-administered questionnaire was distributed to adults aged 18 and older to assess their knowledge and attitudes towards epistaxis first aid. The questionnaire covered demographics, common causes of epistaxis, and appropriate first aid measures. Data were analyzed using descriptive statistics and inferential tests, including chi-square and binary logistic regression analyses, to identify any associations between knowledge and demographic factors.

Results

A total of 370 participants were surveyed. While most respondents (n=325, 87.8%) recognized the importance of first aid for epistaxis, only (n=223, 60.4%) knew that pressure should be applied to the nose to stop bleeding, and only (n=130, 35.1%) were aware of the correct location for applying pressure. The average knowledge score was 3.1 out of 6, revealing substantial gaps in public understanding. Demographic factors, such as age, gender, and education level, did not show significant associations with knowledge scores.

Conclusion

The study reveals that there are significant gaps in knowledge regarding epistaxis first aid in Al-Qatif. Public health initiatives focused on educating the population about proper management techniques are crucial to reducing the risk of complications.

## Introduction

Of the challenging acute presentations faced by otorhinolaryngologists, epistaxis is a potentially life-threatening condition accounting for 60% of incidence in the American population, which may vary in severity, ranging from moderate to severe, imposing great stress on both physicians and patients [1]. To clarify the burden of epistaxis, in the United Kingdom, 25,000 cases of epistaxis are encountered per year, with an average of 1,400 admissions per month [2]. Existing data suggest matching statistics regarding epistaxis events up to 49% among the general population of the Kingdom of Saudia Arabia (KSA) with 35.5% in the children measured as 0.98% of all the emergencies across the country [3-5].

Considering age distribution, fewer cases were reported among newborns and infants, but bimodal distribution has been observed presenting earlier at the age of 2-10 years and 50-70 years with male predominance, which are attributed to several local factors, including nose pricking, upper respiratory infections/allergies, dryness of the air, foreign body and septal deformities [6]. Treatment-resistant recurrent episodes of epistaxis may enquire about underlying systemic associations, especially related to blood dyscrasias, anti-coagulation, and coagulopathy, which could be managed as in-patient [7].

“First aid” in epistaxis has been described as emergency therapy that controls bleeding, preventing its progression to a worsening event before evaluation in the healthcare setting, empowering its key role in harnessing mortality [8]. Except for certain cases, most are self-limiting, requiring rare medical advice, while the other cases could be managed with pressure application or nasal packing, strengthening the need for its awareness and related practice among the general population [9].

Previous studies strongly suggest gaps in the level of knowledge, attitudes, and practices toward first aid measures among the general population of KSA, without which complications may arise, leading to mortality. Poor knowledge and practices among parents about epistaxis, its causative factors, and first aid measures implied to stop bleeding at home were postulated in another prospective cross-sectional study of Nigeria, fostering the need for awareness among parents [10]. A recent cross-sectional study conducted in Makkah recognized notable gaps in knowledge status regarding epistaxis first aid with poor knowledge found among 37.1% of people [11]. Coherent results were also observed in the Jazan region, KSA, where 49.7% of people had poor knowledge and practices, as revealed by a data-based study [12].

In contrast, the population of Riyadh, KSA, showed good knowledge and better practices, where 81% of people correctly identified the first step in the management of epistaxis, as highlighted by Mohammad et al. [13]. A survey conducted in Taif, KSA evaluated the awareness level among parents for providing first aid to children with epistaxis. Accordingly, only 30.9% of parents carried satisfactory knowledge about first aid, focusing on the relative gaps in understanding commonly faced emergencies at home [14]. Hence, teaching and training may prove crucial for significant outcomes.

While intervening in an epistaxis emergency, physicians may acquire an in-depth understanding of history, examination, and investigation to establish the cause, which is not feasible by the general population with a goal of approaching the definitive care in a healthcare setting; however, encountering this in public places may provoke a stressful event [15]. Such aspects could be avoided by having fundamental knowledge and practicing first aid maneuvers among non-health professionals, a significant preemptive move towards mitigation of the significant burden [16]. Therefore, the current study was conducted to evaluate the knowledge and attitude of the general population of Al-Qatif towards epistaxis first aid. Concerned with the previous data, researchers focused on the knowledge without measuring the sociodemographic factors related to attitude, we explored knowledge and attitude domains among the general population.

To the best of our knowledge, this is the first study to assess knowledge and attitude towards epistaxis first aid in the Al-Qatif region, KSA, potentially offering a basis for comparative analysis to improve related strategies. Unveiling the misconceptions and malpractice during the management of epistaxis, the current study may be able to design a targeted plan for educational campaigns. Enhanced awareness about first aid practices during an episode of epistaxis may alleviate potential life-threatening events with an ultimate sustained qualitative life.

## Materials and methods

Methods

This study employed a cross-sectional survey design to gather data from the general population of Qatif, Saudi Arabia, aged 18 years and older, between November and December 2024.

Inclusion and exclusion criteria

Participants were recruited randomly. The inclusion criteria for this study were individuals aged 18 years or older who resided in Qatif, Saudi Arabia. Participants needed to be part of the general population and willing to provide informed consent to take part in the study. The exclusion criteria encompassed individuals under 18 years of age, non-residents of Qatif, and those who either declined to participate or submitted incomplete responses to the questionnaire.

Developing the questionnaire

The questionnaire was designed in two sections based on previous research [11]. The first section aimed to gather demographic information, including participants' sex, age, residence, marital status, educational level, career, and monthly income. These variables were chosen to capture key socio-economic factors that may influence knowledge and awareness. The second section focused on assessing participants' knowledge of epistaxis through a knowledge score derived from six carefully crafted questions. These questions were designed to evaluate participants' understanding of the causes, symptoms, and treatment of epistaxis. Correct responses were scored to provide a comprehensive knowledge score for each participant.

Consent

Ethical approval for the study was obtained from the Research Ethics Committee of the University of Jeddah, Saudi Arabia (IRB Registration No: HAP-02-J-094). Prior to completing the questionnaire, participants were asked to provide informed consent. This consent was provided electronically at the beginning of the survey. The consent adhered to international guidelines, Participants were fully informed about the study’s objectives, procedures, and their right to decline or withdraw from the study at any time without consequence.

Statistical methods

Data analysis was performed using Statistical Product and Service Solutions (SPSS, version 26; IBM SPSS Statistics for Windows, Armonk, NY). For knowledge assessment, participants earned one point for each correct answer, while incorrect responses received zero points. The total knowledge score was calculated by summing the scores across all questions, yielding a range from 0 to 6 points. The frequency and percentage of responses for each question were determined. Additionally, the mean and standard deviation (SD) were computed for each question to offer a detailed descriptive analysis. To assess the effect of different variables on the knowledge score, binary logistic regression analysis was utilized.

## Results

Characteristics

The demographic characteristics of the study cohort (n=370) indicate a higher prevalence of male participants (n=234, 63.2%) compared to females (n=136, 36.8%). The age distribution is predominantly skewed toward older age groups, with 35.4% aged >50 years and 25.1% aged 40-49 years. Meanwhile, smaller proportions are represented in the 19-29 years (n=65, 17.6%), 30-39 years (n=45, 12.2%), and <18 years (n=36, 9.7%) categories. Urban residency predominates, with 271 (73.2%) residing in cities compared to 29 (26.8%) in villages. Marital status reveals that the majority are married (n=272, 73.5%), followed by single individuals (n=94, 25.4%), with widowed and divorced participants collectively accounting for four (1%). Educational attainment shows that 187 (50.5%) have completed postgraduate education, 164 (44.3%) have attained high school qualifications, and lower proportions hold intermediate (n=13, 3.5%) or primary school (n=6, 1.6%) education levels. In terms of occupational status, non-healthcare workers comprise the majority (n=219, 59.2%), followed by unemployed individuals (n=125, 33.8%) and healthcare workers (n=26, 7%). Monthly income distribution highlights a considerable segment of participants reporting no income (n=125, 33.8%); 101 (27.3%) earn >15,000; 92 (24.9%) earn between 5,000 and 15,000; and 52 (14.1%) earn <5,000 (Table 1).

**Table 1:** Characteristics of the participants.

Variables	n	%
Sex		
Female	136	36.8
Male	234	63.2
Age		
<18	36	9.7
19-29	65	17.6
30-39	45	12.2
40-49	93	25.1
>50	131	35.4
Residence		
Village	99	26.8
City	271	73.2
Marital status		
Widow	2	0.5
Single	94	25.4
Married	272	73.5
Divorced	2	0.5
Educational level		
Primary school	6	1.6
High school	164	44.3
Postgraduate	187	50.5
Intermediate school	13	3.5
Career		
Healthcare worker	26	7
Non-healthcare worker	219	59.2
Unemployed	125	33.8
Monthly income		
>15000	101	27.3
5000-15000	92	24.9
<5000	52	14.1
Nothing	125	33.8

Association between demographic variables and history of epistaxis

The relationship between various demographic variables and the occurrence of epistaxis was assessed using chi-square tests. Overall, 23 participants had a history of epistaxis. The analysis revealed no statistically significant associations across the examined factors. Regarding sex, 10 (7.4%) females and 13 (5.6%) males reported a history of epistaxis (p=0.49). Age stratification showed that individuals <18 years had the highest prevalence of epistaxis (n=4, 11.2%), while the 30-39 group exhibited the lowest prevalence (n=2, 4.5%), though the differences were not significant (p=0.696). Residence showed a trend toward higher epistaxis rates among city dwellers (n=20, 7.4%) compared to village residents (n=3, 3.1%), but this was not statistically significant (p=0.125).

Marital status indicated a slightly higher prevalence among single individuals (n=8, 8.6%) compared to married participants (5.6%), with no cases reported among widowed or divorced individuals (p=0.719). Educational level showed no significant association, though primary school attendees had the highest prevalence (n=1, 16.7%) (p=0.463). Among occupational groups, unemployed participants exhibited a higher prevalence (n=17, 13.6%) compared to healthcare workers (n=2, 7.7%) and non-healthcare workers (n=4, 1.9%), but the difference was non-significant (p=0.653). Monthly income levels did not correlate significantly with epistaxis prevalence, as the rates ranged from 4 (4.4%) to 4 (7.7%) across income categories (p=0.809). Overall, no demographic variable showed a significant association with a history of epistaxis in this cohort (Table 2).

**Table 2:** Characteristics of the participants according to previous epistaxis.

Variables	Ever had epistaxis				P-value
	No (n, %)		Yes (n, %)		
Sex					0.49
Female	126	92.6	10	7.4	
Male	221	94.4	13	5.6	
Age					0.696
<18	32	88.8	4	11.2	
19-29	62	95.3	3	4.7	
30-39	43	95.5	2	4.5	
40-49	88	94.6	5	5.4	
>50	122	93.1	9	6.9	
Residence					0.125
Village	96	96.9	3	3.1	
City	251	92.6	20	7.4	
Marital status					0.719
Widow	2	100	0	0	
Single	86	91.4	8	8.6	
Married	257	94.4	15	5.6	
Divorced	2	100	0	0	
Educational level					0.463
Primary school	5	83.3	1	16.7	
High school	152	92.6	12	7.4	
Postgraduate	177	94.6	10	5.4	
Intermediate school	13	100	0	0	
Career					0.653
Healthcare worker	24	92.3	2	7.7	
Non-healthcare worker	215	98.1	4	1.9	
Unemployed	108	86.4	17	13.6	
Monthly income					0.809
>15000	95	94.1	6	5.9	
5000-15000	88	95.6	4	4.4	
<5000	48	92.3	4	7.7	
Nothing	116	92.8	9	7.2	

Knowledge assessment

**Figure 1:**
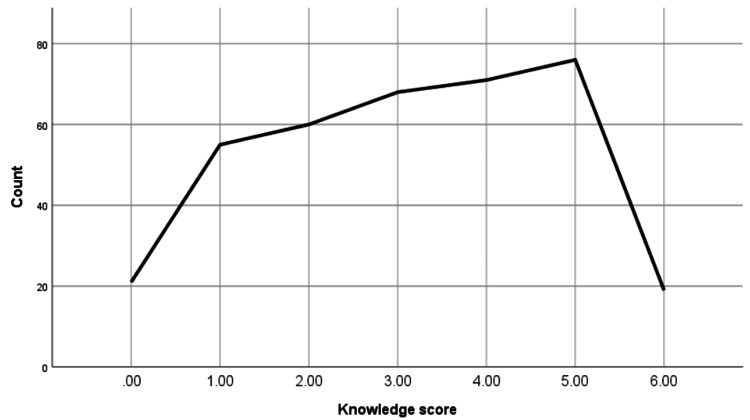
Knowledge score.

The knowledge assessment on epistaxis first-aid measures revealed varying levels of understanding among participants (Figure 1). The vast majority (n=325, 87.8%) recognized the importance of first-aid measures, with a mean score of 0.88 (SD=0.32). However, knowledge of specific measures was inconsistent. While 223 (60.4%) correctly identified that applying pressure on the nose can stop epistaxis (mean=0.61, SD=0.48), only 130 (35.1%) knew the correct part of the nose to apply pressure (mean=0.35, SD=0.47).

Similarly, only 77 (20.8%) were aware of the correct duration for applying pressure (mean=0.21, SD=0.41), and 187 (50.5%) identified the best position to stop epistaxis (mean=0.51, SD=0.50). Regarding the timing of seeking emergency care, 214 (57.8%) answered correctly (mean=0.58, SD=0.49). The overall mean knowledge score was 3.1 (SD=1.7), highlighting substantial gaps in practical knowledge of epistaxis management among the participants (Table 3).

**Table 3:** Knowledge score for epistaxis.

Questions	Correct (%)	Incorrect (%)	Mean (SD)
Do you think first-aid measures are important for epistaxis?	87.8	12.2	0.88 (032)
What is the best position to stop epistaxis?	50.5	49.5	0.51 (0.50)
Applying pressure on the nose can stop epistaxis?	60.4	39.5	0.61 (0.48)
Which part of the nose will you apply pressure during epistaxis?	35.1	64.9	0.35 (0.47)
How long will you apply pressure on your nose?	20.8	79.2	0.21 (0.41)
What will be the right time to go to the ER?	57.8	42.2	0.58 (0.49)
Total			3.1 (1.7)

Association between demographic variables and knowledge scores on epistaxis management

The relationship between demographic characteristics and knowledge scores on epistaxis management was examined. Participants were categorized into two groups: poor knowledge (scores 0-3) and good knowledge (scores 4-6). No statistically significant associations were found between knowledge scores and any of the demographic variables assessed. Regarding sex, 70 (51.4%) females and 134 (57.2%) males were categorized as having poor knowledge (p=0.414). Across age groups, individuals aged 30-39 showed the highest proportion of good knowledge (n=26, 57.8%), while those >50 years had the lowest (n=51, 38.9%) (p=0.36). Residence showed no significant impact, with poor knowledge observed in 51 (51.5%) of participants from villages and 153 (56.4%) from cities (p=0.49).

Marital status also demonstrated no significant association, although 47 (50%) single participants exhibited good knowledge, compared to 119 (43.8%) married individuals (p=0.172). Educational level did not significantly influence knowledge scores, with postgraduate participants showing slightly higher good knowledge (n=82, 43.9%, p=0.871). Career and income also showed no significant differences. Poor knowledge was more frequent among healthcare workers (61.5%) and those earning no income (n=74, 59.2%), while good knowledge was slightly higher among those earning >15,000 (n=52, 51.5%, p=0.426, and p=0.329, respectively) (Table 4).

**Table 4:** Characteristics of the participants according to the level of knowledge.

Variables	Knowledge score				P-value
	Poor (0-3)		Good (4-6)		
	(n, %)		(n, %)		
Sex					0.414
Female	70	51.4	66	48.6	
Male	134	57.2	100	42.8	
Age					0.36
<18	19	52.7	17	47.3	
19-29	34	52.3	31	47.7	
30-39	19	42.2	26	57.8	
40-49	52	55.9	41	44.1	
>50	80	61.1	51	38.9	
Residence					0.49
Village	51	51.5	48	48.5	
City	153	56.4	118	43.6	
Marital status					0.172
Widow	2	100	0	0	
Single	47	50	47	50	
Married	153	56.2	119	43.8	
Divorced	2	100	0	0	
Educational level					0.871
Primary school	4	66.6	2	33.4	
High school	88	53.6	76	46.4	
Postgraduate	105	56.1	82	43.9	
Intermediate school	7	53.8	6	46.2	
Career					0.426
Healthcare worker	16	61.5	10	38.5	
Non-healthcare worker	118	53.8	101	46.2	
Unemployed	75	60	55	40	
Monthly income					0.329
>15000	49	48.5	52	51.5	
5000-15000	54	58.6	38	41.4	
<5000	27	51.9	25	48.1	
Nothing	74	59.2	51	40.8	

Factors affecting the knowledge score

The results indicated no statistically significant associations for any of the variables analyzed. Gender showed an odds ratio (OR) of 0.927 (95% CI: 0.812-1.126, p=0.762), suggesting no significant difference in knowledge between males and females. Similarly, individuals aged >39 years had a slightly lower likelihood of good knowledge (OR: 0.823, 95% CI: 0.762-0.982, p=0.454), although this was not statistically significant.

Participants residing in cities had an OR of 0.972 (95% CI: 0.922-1.246, p=0.256), while married individuals demonstrated an OR of 0.978 (95% CI: 0.967-1.158, p=0.342), neither showing significant associations with knowledge levels. Educational level and career also showed no significant impact, with postgraduates having an OR of 0.856 (95% CI: 0.952-1.181, p=0.259) and healthcare workers an OR of 0.982 (95% CI: 0.915-1.140, p=0.125) (Table 5).

**Table 5:** The association between multiple variables and the knowledge score.

Dimension	Variable	p-value	OR	95 % CI for OR
Knowledge	Gender (Males)	0.762	0.927	(0.812, 1.126)
	Age (>39)	0.454	0.823	(0.762, 0.982)
	Residence (city)	0.256	0.972	(0.922, 1.246)
	Marital status (married)	0.342	0.978	(0.967, 1.158)
	Educational level (Postgraduate)	0.259	0.856	(0.952, 1.181)
	Career (healthcare)	0.125	0.982	(0.915, 1.140)

Source of knowledge

**Figure 2:**
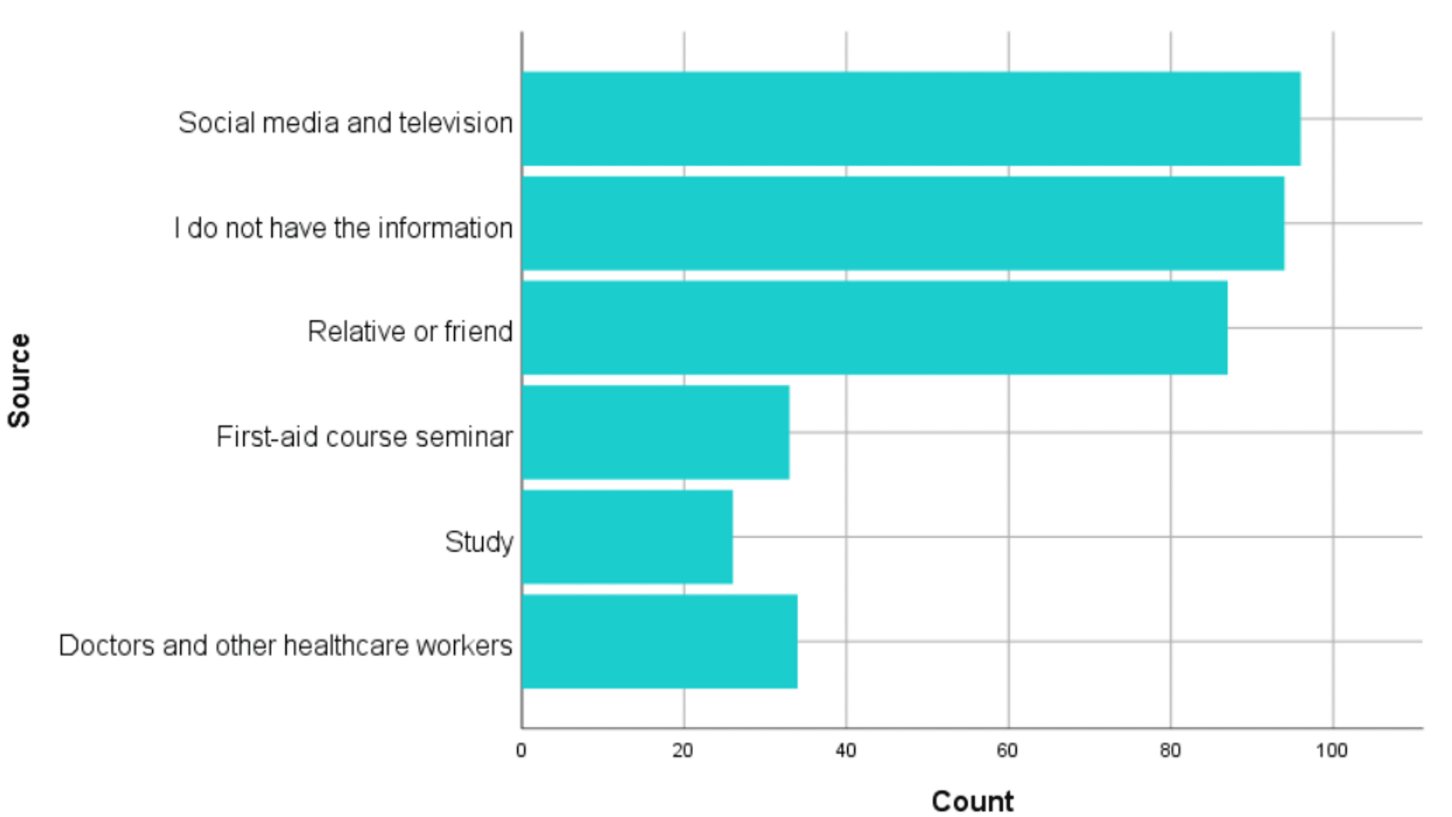
Sources of information regarding epistaxis.

The sources of knowledge regarding epistaxis management among participants were diverse. Social media and television emerged as the most common sources, reported by 96 (25.9%) respondents, followed closely by those who indicated that they lacked any relevant knowledge (n=94, 25.4%). Friends and relatives were the third most frequent source, accounting for 87 (23.5%) responses. Formal education contributed to a smaller proportion, with 34 (9.2%) citing healthcare practitioners and 33 (8.9%) attributing their knowledge to first aid training courses. Academic studies were the least reported source, with only 26 (7%) of participants identifying it as their primary means of acquiring knowledge about epistaxis management (Figure 2).

## Discussion

Owing to the fatal consequences due to epistaxis, awareness among the general population is desirable, which may prevent the potentiation of life-threatening situations by providing first aid before approaching healthcare settings. We assessed the general knowledge of our population regarding epistaxis first aid through this cross-sectional study. To our results, although substantial gaps in knowledge regarding epistaxis first aid were measured among our population, especially regarding the correct part of the nose to apply pressure and the duration of pressure, most people were aware of applying pressure over the nose as the measure of first aid for epistaxis. The people with higher educational levels showed good knowledge in managing epistaxis, while poor knowledge was more among healthcare workers. Social media and television were the most common sources for knowledge regarding epistaxis first aid measures, with few people achieving it from academic studies.

From the findings, most participants (87.8%) fostered a better understanding of epistaxis first aid, and an influence might be established with higher education and healthcare providers based on experiences through various modalities. Almost 67% of participants constituted a better knowledge level, which is congruent with the current analytical outcomes [17]. Awareness regarding first aid implied in epistaxis was good among 74.6% of people, as demonstrated by an observational study, further strengthening our findings [18]. Compared to this study, 82% of the people were not familiar with the term “epistaxis,” while 66% of participants considered nose compression as the cause of epistaxis [16]. Good awareness regarding epistaxis and its first aid step was recorded in only 19.4% of participants, as revealed by the study, which is also comparable to our outcomes, suggesting better acknowledgment in our sample. Disclosure of such outcomes could be associated with educational background and sociodemographic and socioeconomic factors, which must be signified at the societal level [19].

The application of pressure as the fundamental means of stopping nasal bleeding by our population (60.4%) was found coherent with results by Alkhalaf et al.; however, knowledge regarding the ideal technique of compression and duration of compression was lacking, emphasizing the recognition of factors in its background [20]. The correct part of the nose for applying pressure was recognized by 35.1%, and only 20.8% of people revealed the exact duration of compression in our study, highlighting an insignificant role of higher education because 50.5% of our participants are postgraduates. Poor knowledge regarding the site and duration of pressure was also experienced by health professionals in a recent study with a more negative correlation among emergency physicians [21]. Our sample constitutes only 7% of health-related members, which could be attributed to poor knowledge about the steps of first aid in epistaxis.

Coinciding results were also shown by Alasiri et al. as only 15.5% of individuals were aware of the steps regarding the site and duration of pressure during epistaxis episodes. Training sessions and conferences with prior experience were the main reasons among people familiar with the first aid steps [3]. Relative to this, some studies stratified poor acquisition of practical knowledge among healthcare providers, with only 30% correctly demarcating the site of compression, which further emphasizes the need for training and practice [22]. Poor awareness without prior experience in providing first aid in epistaxis may enhance the fatalities due to increased risk of blood loss, recurrence, and prolonged hospitalization [23].

Significant gaps in practical implications of knowledge of healthcare workers have been highlighted, which fail to provide focused techniques and are challenged by recurrent episodes of epistaxis enhancing mortality. A study by Sowerby et al. evaluated the first aid steps of healthcare workers and identified deficient areas that need improvement through structured training [24]. Boldes et al. also marked poor practical steps in epistaxis first aid, an alarming situation having a direct impact on patient's health [21]. Training sessions and international guidelines must be exercised by healthcare professionals, which may enhance its implications in terms of patient positioning and applying pressure at the correct site for a certain duration.

We found a slightly good knowledge regarding epistaxis first aid among 43.9% of postgraduates, which could be attributed secondary to the direct impact of educational level for having general awareness. Surprisingly, a recent study postulated a negligible role of educational standards in the improvement of epistaxis first aid measures, rather prior experience and practice of being trained had a great impact [25]. Teachers' ability in first aid was assessed, which resulted in only one-third of teachers being able to manifest first aid correctly, of which mostly had a history of prior experience with such measures [3]. Similar findings were also explored in another study, with poor performance in treating epistaxis, following first-aid steps by teachers [26]. The lack of prior experience, health education, and poor attitude could be the possible reasons for not having practical knowledge of such steps, rather than prioritizing the role of educational standards [16].

Despite holding a wide variation, the general population (6%) had poorer knowledge than medical professionals (80%) possibly due to medical affiliation, which may offer consistent exposure to events of epistaxis, along with teaching and training sessions [1]. Poor knowledge level among health professionals (61.5%) in our study could be attributed to their only 7% participation than non-healthcare workers (59.2%). Although consistent with the current findings, still a comparable proportion of medical professionals (41.3%) had good knowledge regarding first aid in epistaxis as revealed by Khalid et al. [27].

Published data highlighted the significant role of social media and television in acquiring information for managing epistaxis by most of the population than academic studies, which is coherent with our findings [1]. Of the total population, only 23.33% of individuals preferred academic studies as a source for epistaxis in a study by Khalid et al., setting a similar trend with the current results (7%). Compared to the general people in this study, Khalid et al. conducted a study among medical students, a possible reason behind this proportional difference [27]. Another cross-sectional study revealed poor knowledge among 55.7% of health workers with academic studies as the source of only 38.3% of people, which is in line with our results [18]. A study postulated a slightly improved status of awareness among the general population (36%), not better than the medical community (40-50%) due to the failure of in-depth understanding of practical implications and direct participation in managing epistaxis [1].

The strengths of our study must be highlighted. An in-depth understanding of potential gaps has been determined while managing epistaxis among the general population, which may guide the formulation of the national standards and their implications in the form of setting seminars and awareness sessions. Selecting an optimum population size may avoid any health-related damage to the public by producing false results. Our study has certain limitations, which must be acknowledged to address gaps. As the study participants mostly belong to Saudi Arabia, generalizability issues may arise, and results could not reflect the awareness levels beyond Saudi populations. Bias while collecting web-based data could not be neglected which may hinder its broader representation.

## Conclusions

In conclusion, while the study reveals a general awareness of epistaxis first aid measures, substantial gaps in knowledge remain, particularly regarding the correct application site and duration of pressure. The findings emphasize the need for targeted educational interventions, especially among healthcare workers, to improve practical skills. Raising awareness through training and guidelines could potentially reduce the risk of complications and fatalities associated with epistaxis.

## Appendices

The Survey Questions

Part 1: Demographic Data

Q1 Are you living in the Qatif region?

o Yes

o No

Q2 Gender?

o Female

o Male

Q3 Age?

o <18

o 19-29

o 30-39

o 40-49

o 50 or above

Q4 Living in?

o City

o Village

Q5 Marital status?

o Single

o Married

o Divorced

o Widow

Q6 Educational level?

o Primary school

o Intermediate school

o High school

o Postgraduate

Q7 Career?

o Healthcare worker

o Non-healthcare worker

o Unemployed

o Other

Q8 Monthly income?

o <5000 SR

o 5000-15000 SR

o >15000 SR

o No income

Part 2: Knowledge, Attitude, and Practice of First-Aid Management of Epistaxis

Q1 Do you suffer from epistaxis?

o Yes

o No

Q2 Do you think first-aid measures are important for epistaxis?

o Yes

o No

o Do not Know

Q3 What is the best position to stop epistaxis?

o Lie down with your feet up

o Tilt the head forward

o Tilt the head back

o Do not know

Q4 Will applying pressure on the nose stops epistaxis?

o Yes

o No

o Do not know

Q5 Which part of the nose will you apply pressure during epistaxis?

o The lower part (cartilage)

o Upper part (bone)

o Do not know

Q6 How long will you apply pressure on your nose?

o 5 minutes

o 6-10 minutes

o 11-15 minutes

o 16-20 minutes

o Do not know

Q7 What will be the right time to go to the ER?

o Once the epistaxis happen

o If the bleeding does not stop after more than 20 minutes

o If the bleeding does not stop after more than 40 minutes

o If the bleeding does not stop after more than 60 minutes

o I will not go to the ER at all

o Do not know

Q8 Source of information?

o Relative or friend

o Social media and television

o First-aid course seminar

o Doctors and other healthcare workers

o I do not have the information

o Study
